# Feeding with 4,4′-diaponeurosporene-producing *Bacillus subtilis* enhances the lactogenic immunity of sow

**DOI:** 10.1186/s12917-023-03846-3

**Published:** 2023-12-19

**Authors:** Peng Liu, Qi Zhang, Chengjie Yang, Xiuyu Wang, Yuchen Li, Jianda Li, Qian Yang

**Affiliations:** MOE Joint International Research Laboratory of Animal Health and Food Safety, College of Veterinary Medicine, Nanjing Agricultural University, Jiangsu, 210095 PR China

**Keywords:** Congenital lactogenic immunity, 4,4′-diaponeurosporene-producing *Bacillus subtilis* (*B.S*-Dia), Cytokines, Lysozyme and lactoferrin, PEDV

## Abstract

Specific antibodies produced sow by oral porcine epidemic diarrhea virus (PEDV) vaccines would transfer to newborn piglets via colostrum, and it is an effective strategy to prevent porcine epidemic diarrhea (PED). However, there is a lag in the development of corresponding vaccines due to the rapid mutation of PEDV, which could increase the difficulty of PED prevention and control in pig farms. Hence, congenital lactogenic immunity was assessed by feeding 4,4′-diaponeurosporene-producing *Bacillus subtilis* (*B.S*-Dia) to sow on the 80th day of gestation in order to protect newborn piglets from PEDV infection. Firstly, we found that the quantities of T lymphocytes and monocytes in the blood and colostrum after oral administration of *B.S*-Dia were significantly increased as observed by flow cytometry, whereas the proliferative activity of T lymphocytes in colostrum was also markedly increased. Furthermore, enzyme-linked immunosorbent assay (ELISA) results revealed that levels of TGF (Transforming growth factor) -β, Interleukin (IL) -6, lysozyme and lactoferrin were significantly increased. Finally, it was found in the piglets’ challenge protection test that offspring pigs of the sows feeding *B.S*-Dia during pregnancy did not develop diarrhea symptoms and intestinal pathological changes at 48 h after infection with PEDV, and PEDV load in the jejunum and ileum was significantly reduced, but offspring pigs of the sows taking orally PBS during pregnancy developed pronounced diarrhea symptoms and extensive PEDV colonization was noted both in the jejunum and ileum. In summary, sow by oral administration of *B.S*-Dia substantially increased congenital lactogenic immunity, thereby preventing newborn piglets from being infected with PEDV.

**Supplementary Information:**

The online version contains supplementary material available at 10.1186/s12917-023-03846-3.

## Introduction

Porcine epidemic diarrhea (PED) is an infectious gastrointestinal disease caused by the porcine epidemic diarrhea virus (PEDV) and is associated with high morbidity and mortality which can reach 90% in newborn piglets. Most currently used vaccines for PED prevention are administered by intramuscular injection; however, these vaccines are unable to induce sufficient immune protection due to the imperfect development of the immune system of newborn piglets [1]. Thus, recent studies have investigated the possibility of protecting piglets from PEDV infection by immunizing the sow, through the use of refeeding and oral administration of PEDV vaccines [2, 3]. PEDV-specific antibodies in the sow’s colostrum play a major role in the efficacy of lactogenic immunity-induced PEDV vaccines [4]. For instance, Saif et al. reported that administration of oral PEDV to pregnant sows could markedly increase the concentration of specific antibodies and the number of IgA^+^ B lymphocytes in the colostrum, leading to significantly reduced mortality from PEDV infection in piglets [2]. Additionally, pregnant sows receiving an oral attenuated PEDV vaccine also showed elevated levels of PEDV-specific antibodies in the colostrum [5].

The oral administration of PEDV vaccines to sows induces the production of IgA plasmablasts in the intestine. These cells can then migrate to the mammary gland through the gut-mammary gland-SIgA axis, resulting in the secretion of dimeric IgA antibodies into the colostrum to form specific SIgAs [6]. Vaccination remains the most promising and effective way to prevent and control PEDV. However, effective vaccines for epidemic PEDV strains in swine farms are still under development [7]. Studies have also demonstrated difficulties in cross-protection against different PEDV strains [8, 9]. Hence, strategies to improve the congenital lactogenic immunity of sows to protect piglets from PEDV infection may be effective.

*Bacillus subtilis* (*B.S*) is a commonly-used probiotic that can be used as an additive in animal feed [10]. Carotenoid displays unique biological functions and can act as a potent mucosal immune enhancer. 4,4′-diaponeurosporene-producing *Bacillus subtilis* (*B.S*-Dia) was obtained as previously described [11]. A previous study in our laboratory found that mice fed *B.S*-Dia exhibited improved congenital intestinal immunity, and were able to effectively resist *Salmonella* infection [12]. In current study, the effects of feeding *B.S*-Dia to sows on congenital lactogenic immunity were investigated. The challenge protection test was used to verify whether the innate lactogenic immunity acquired by the piglets could effectively resist to the PEDV infection, to provide another pathway for the effective prevention and control of PED.

## Materials and methods

### Animals

In this study, the sows were purchased from Hongsong pig farm in Gaochun City, Jiangsu Province, China. Tests for antibodies against PEDV, porcine reproductive and respiratory syndrome virus (PRRSV), porcine respiratory coronavirus (PRCV), transmissible gastroenteritis virus (TGEV), pseudorabies virus (PRV), and porcine circovirus 2 (PCV2) were negative.

### Cells and virus strains

Vero E6 cells and PEDV Zhejiang 08 (epidemic strain) were obtained from Dabei Agricultural Animal Medicine Research Center. Swine testicular (ST) cells were stored in the laboratory. TGEV (SHXB) was provided by the Jiangsu Provincial Academy of Environmental Science (JAAS).

### Experimental design

**Fig. 1:**
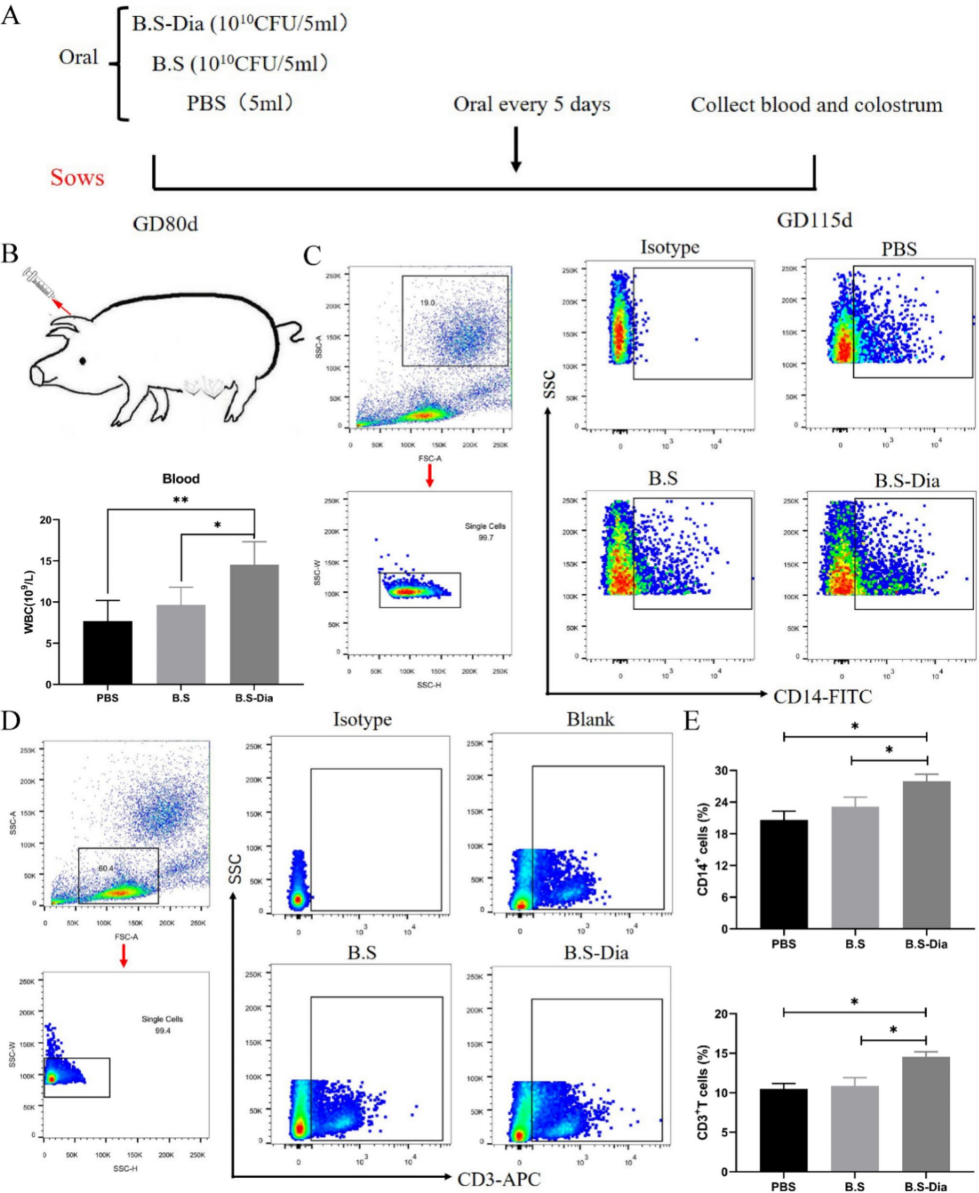
**(A)** Schematic diagram showing oral administration of *B.S-*Dia, *B.S*, and PBS to sows; **(B)** Blood was collected from the auricular vein and analyzed by an automated cell analyzer, n = 5; **(C-D)** CD14^+^ monocytes and CD3^+^T lymphocytes in blood were detected by flow cytometry, n = 5; **(E)** The number of positive cells in blood was counted. Differences between the different treatment groups **(B, E)** were analyzed by one-way ANOVA, n = 5, *P < 0.05

Thirty third-parity sows (day 80 of gestation) were randomly selected from the pig farm and were divided into three groups, namely, the *B.S*-Dia, *B.S*, and PBS groups. In the *B.S*-Dia group, of 5 mL *B.S*-Dia (1 × 10^10^ CFU/ mL) was fed, while in the B.S group, 5 mL of Bacillus subtilis (1 × 1010 CFU/ mL) was given and the PBS group received an oral dose of 5 mL of phosphate-buffered saline (PBS). The sows were all fed on day 80 of gestation (GD80) and received further oral doses every 5 days until delivery (GD115) (Fig. 1A). Mammary gland biopsies were collected after delivery [13]. The mammary glands were divided into different parts, namely, the base area (BA), the central area of the upper body (CAUB), and the area surrounding the gland cistern (ASGC) located at the base of the teat [14]. Tissues located in BA were collected. Blood samples (10 mL) from the auricular vein were collected into glass tubes containing heparin sodium.

The piglets were free to suck breast milk after birth. At 5 days of age, 5 piglets from each group were randomly selected and orally infected with PEDV Zhejiang 08 strain (TCID_50_ = 3). All the piglets were euthanized 48 h after infection, with the time of animal execution determined according to a previous study [15]. Euthanasia of piglets was performed applying an overdose of pentobarbital sodium administered by a veterinarian assisted by properly trained personnel, and dosages through intravenous injection was 100 mg/kg [16, 17]. Meanwhile, verification that death has occurred was carried out by absence of rhythmic breathing and pupillary reflexes. Fecal samples were collected at 0, 6, 12, 24, 36, and 48 h after infection using a sterile transport swab inserted into the rectal cavity. The swab was gently rotated through 360° several times during sample collection to absorb secretions from the wall of the rectal cavity. The clinical symptoms of the piglets were also observed and blood, duodenum, jejunum, ileum, and mesenteric lymph node samples were collected for analysis. In addition, clinical symptoms were monitored and scored between 0 and 48 h after infection. The specific scoring criteria were as follows: 0, hard shaped stool; 1, soft and shaped feces; 2, semi-solid feces lacking shape; 3, watery diarrhea. After euthanasia, pathological changes, such as transparency, in the intestines were assessed.

### Detection of immune cells in sow blood

One milliliter of whole blood was used for analysis using a fully automatic blood analyzer (BC-5000, Nanjing Baden Medical Co., Ltd., China). The remaining blood was used for the separation of monocytes and lymphocytes. Nine milliliters of lymphocyte separation solution were placed in a sterile 50 mL centrifuge tube, after which an equal volume of anticoagulant was slowly added, and the mixture was centrifuged at 400×g for 25 min. The white layer in the middle was collected and washed twice with RPMI 1640 medium and the collected cells were resuspended 100 µL of PBS. Flow cytometry was used to identify CD3^+^T lymphocytes and CD14^+^monocytes using APC mouse anti-pig CD3ε (catalog number, 561,476, APC mouse anti pig CD3ε, BD, Franklin Lakes, NJ, USA) and rabbit anti-pig CD14 (catalog number, 17000-1-AP, Proteintech, Rosemont, IL, USA) antibodies, respectively.

### Detection of immune cell numbers and lymphocyte proliferation in colostrum

Fifty milliliters of colostrum was collected from sows after delivery. Of this, 20 mL was used for cell separation as previously described [18] with some modifications. Briefly, the colostrum was filtered through a nylon mesh (210 μm) to remove impurities, after which it was centrifuged (340×g, 15 min) and the fat layer and supernatant were discarded. The cell precipitate was then washed with pre-cooled PBS, centrifuged at 340×g for 15 min, and the supernatant was discarded. The cell precipitate was resuspended in 100 µL PBS for the identification of CD3^+^T lymphocytes and CD14^+^monocytes by flow cytometry.

The remaining 30 mL of colostrum was used for lymphocyte isolation using Lactation Lymphocyte Separation Solution (Beijing Solarbio Biotechnology Co., Ltd., China). The cells in the colostrum were isolated after centrifugation (500×g, 10 min) and resuspended in sample dilution buffer to prepare single-cell suspensions (2 × 10^8^‒2 × 10^9^ cells/mL). Then, 5 mL of the separation solution was slowly added to the single-cell suspension, followed by centrifugation at room temperature with a horizontal rotor at 500‒900×g for 20‒30 min. The white lymphocyte layer was carefully aspirated using a pipette and transferred to another sterile 15 mL centrifuge tube and washed with 10 mL of cell washing solution. The cells were then centrifuged at 250×g for 10 min, the supernatant was discarded, and the cells were resuspended in 5 mL of PBS or cell washing solution and centrifuged at 250×g for 10 min. The isolated lymphocytes were labeled with carboxyfluorescein succinimidyl ester (catalog number, 565,082, CFSE, BD) and incubated at 37 °C for 8 min, followed by two washes with RPMI medium. The cells were seeded at a concentration of 1 × 10^6^ cells/well in 12-well plates and stimulated by the addition of phytohemagglutinin (PHA, 50ng/mL) or IL-2 (1000 IU) to stimulate the proliferation of T lymphocytes [19]. After 24 h, the cells were centrifuged at 300×g for 10 min and resuspended in 1 mL of PBS, and the proliferative activity of the lymphocytes was assessed by flow cytometry.

### Detection of cytokines and antiviral proteinsin sow colostrum and blood of piglets

The colostrum was first filtered through a nylon mesh (210 μm) to remove impurities and then centrifuged at 340×g for 15 min at 4 °C. The fat layer and sediment were discarded, and the whey was collected. The whey was then centrifuged at 12,000×g for 30 min to remove the residual fat. The collected whey was assayed using non-specific SIgA, IgG, IL-6, IL-4, IFN-γ, TGF-β, CCL2, lactoferrin, and lysozyme ELISA kits, following the instructions of the manufacturer (Shanghai Huyu Biotechnology Co., Ltd., China).

Blood collected from piglets were centrifuged at 3000×g (10 min) for preparation of serum. Serum was assayed by IL-6, lactoferrin and lysozyme ELISA kits.

### Detection of viral load in piglet feces and tissues

Sterile cotton swabs dipped in the piglet feces were placed in 1.5 mL centrifuge tubes. and added with 500 uL PBS, followed by centrifugation at 12,000×g for 15 min and the supernatants collected. Intestinal tissues were ground and centrifuged, with collection of the supernatants. Aliquots (500 µL) of the supernatants from the feces or intestinal tissues were added to 500 µL of TRIzol reagent, and total cellular RNA was extracted according to the traditional TRIzol method. RNA concentrations were standardized reverse-transcribed to cDNA according to the instructions of the HiScript TM QRT SuperMix kit (Novozymes Biotechnology, Bagsvaerd, Denmark), after which the cDNA was diluted 5-fold with DEPC water and amplified with SYBR Green qPCR Master Mix enzyme (Novozymes Biotechnology). The PEDV load in the feces was determined by absolute quantification using relative quantitative PCR, and the plasmid constructed from the PEDV-N gene sequence was used to construct a standard curve. GAPDH was used as the internal reference and the PEDV-N gene was used as the target gene for amplification. The primer sequences are provided in Table 1.

**Table 1:** The primers used in this study

Gene	Primer forward/Reverse	Length
PEDV-N-F	AAGGCGCAAAGACTGAACCC	20 bp
PEDV-N-R	TGTTGCCATTACCACGACTCC	21 bp
GAPDH-F	TCATCATCTCTGCCCCTTCT	20 bp
GAPDH-R	GTCATGAGTCCCTCCACGAT	20 bp

The viral distribution in the intestine was verified by immunofluorescence assay. Sections were dewaxed and incubated in citric acid-sodium citrate buffer (pH = 6.0) for 15 min for antigen repair, washed with PBS, and treated with dropwise administration of 5% bovine serum albumin for 1 h to block nonspecific antibody binding. This was followed by addition of the anti-PEDV-N antibody (PEDV-N, Medgene Labs, Brookings, SD, USA) [20] after five washes with PBS, and incubated overnight at 4 °C. After further washing with PBS, FITC-labeled goat anti-mouse IgG (catalog number, bs-0296G-FITC, Beijing Boarsen Co., Ltd., China) was added to the sections and incubated for 2 h. The sections were then washed 5 times with PBS and 4’,6-diamidino-2-phenylindole (catalog number, 564,907, DAPI, BD) was added to stain the cell nuclei. The viral distribution in the intestinal samples was then assessed using fluorescence confocal microscopy.

### Detection of antiviral properties of whey

Two methods were used for the processing of whey and Vero E6 cells. 1)The collected whey was mixed with PEDV (104 PFU) and incubated in a 37ºC incubator for 1 h. This was then inoculated into Vero E6 cells at 4ºC and 37ºC for 1 h. 2) The collected whey was mixed with Vero E6 cells and incubated in a 37ºC incubator for 1 h. After incubation, the cells were washed with Dulbecco’s Modified Eagle Medium (DMEM) to remove residual protein. Then, PEDV (10^4^ PFU) was added to the cells at 4ºC followed by incubation at 37ºC for 1 h. After incubation, the cells collected by these methods were washed with DMEM to remove residual virus, followed by the addition of 1 mL DMEM containing 2% fetal bovine serum (FBS) to each well. After 24 h, the supernatants and cell samples were collected for plaque tests or RT-qPCR.

The collected whey was mixed with ST cells incubated in a 37ºC incubator for 1 h. After incubation, the cells were washed with Minimum Essential Medium (MEM) to remove residual protein. TGEV (10^4^ PFU) was then added to the cells at 4ºC and incubated at 37ºC for 1 h. The subsequent steps were the same as above.

### Distribution of CD3^+^T lymphocytes in mammary glands and the piglet jejunum

Immunohistochemical (IHC) staining was used to observe the number of CD3^+^T lymphocytes in the tissues. After fixation in 4% paraformaldehyde, the mammary and jejunal tissues were dehydrated in an alcohol gradient and cleared with xylene, followed by paraffin-embedding and sectioning. After the sections were dewaxed, IHC was performed using the Streptavidin-Biotin Complex (SABC) kit (Bode, Seattle, WA, USA). The dewaxed sections were incubated in citric acid-sodium citrate buffer for 15 min for antigen repair. Endogenous peroxidase was blocked by adding 3% H_2_O_2_ and the sections were incubated for 1 h in a 37 °C incubator, washed 5 times with PBS, followed by 5% bovine serum albumin for 1 h to block non-specific antibody binding. The CD3 antibody (catalog number, bs-10498R, rabbit anti-pig CD3, Beijing Boarsen Co., Ltd.) was titrated after washing 5 times with PBS and incubated overnight at 4 °C. The sections were then incubated withd the corresponding biotinylated secondary antibody and incubated for 1 h. After 5 washes with PBS, SABC was added and incubated for 1 h at 37 °C, with color development by 3,3-diaminobenzidine. Finally, the tissue sections were observed under light microscopy.

## Results

### Effects of oral *B.S*-dia on cell numbers and proliferation in sow blood and colostrum

To explore the potential effects of oral *B.S*-Dia to sow on the number of white blood cells (WBCs), blood was collected from the auricular veins of the sows and analyzed by an automated cell analyzer (Fig. 1B). The results indicated that oral administration of *B.S*-Dia (average = 14.6) could significantly increase the quantity of WBCs in the blood compared to the *B.S* (average = 9.7) and PBS (average = 7.7) groups (Fig. 1B). Monocytes and T lymphocytes form the major WBC components and play a key role in viral infection, so the quantities of monocytes and T lymphocytes in blood were measured by flow cytometry. The results showed that oral *B.S*-Dia led to significantly increased numbers of both monocytes and T lymphocytes in the sows’ blood compared to the other groups (*P* < 0.05) (Fig. 1C-E).

**Fig. 2:**
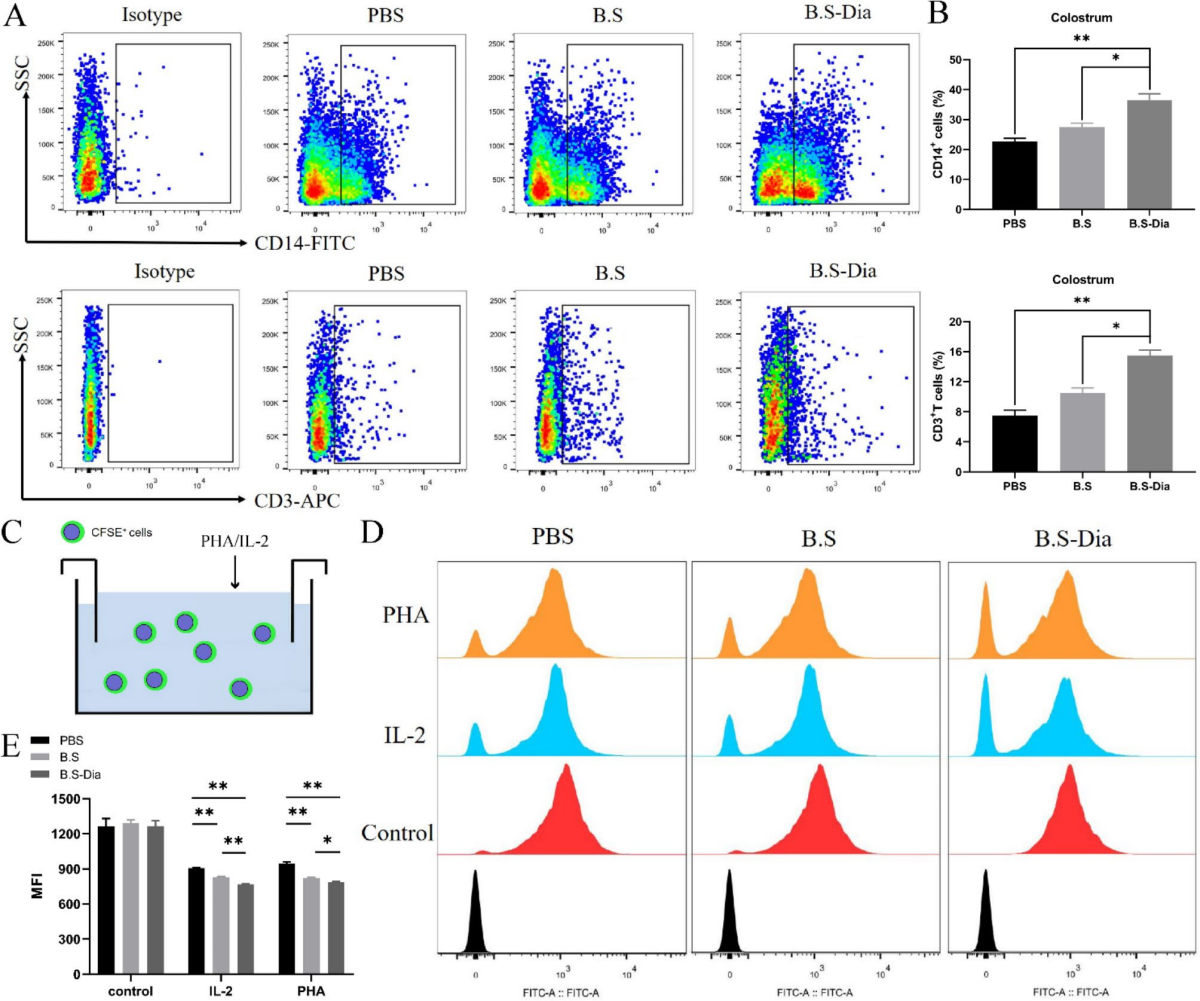
**(A-B)** The number of CD14^+^ monocytes and CD3^+^T lymphocytes in colostrum were detected and counted, and significant differences between the different treatment groups were analyzed by one-way analysis of variance, n = 5, *P < 0.05, **P < 0.01; **(C-E)** Detection of lymphocyte proliferation. Significant differences between different treatment groups **(B, E)** were analyzed by one-way ANOVA, n = 5, *P < 0.05, **P < 0.01

Part of immunocytes in the blood enter in the mammary gland. The IHC showed that significant increases in the number of T lymphocytes in the mammary glands of the *B.S*-Dia group (Supplementary Fig. 1). Furthermore, immune cells in the breast were finally transported into the colostrum, and thus flow cytometry was used to investigate changes in the numbers of monocytes and T lymphocytes in the colostrum. The results revealed that compared with the PBS group, the numbers of monocytes (average = 36.5%) and T lymphocytes (average = 14%) in the colostrum of sows after oral *B.S*-Dia were significantly increased, in comparison with the *B.S* and PBS groups (Fig. 2A-B). In addition to the cell number, the proliferation of T lymphocytes has a critical role in the immune defense of piglets. Compared with the *B.S* group (PHA: average = 821; IL-2: average = 827) and the PBS group (PHA: average = 946; IL-2: average = 907), the fluorescence intensity of T lymphocytes (PHA: average = 788; IL-2: average = 769) was significantly decreased (Fig. 2C-E) in the *B.S*-Dia group, indicating increased proliferation as the fluorescence intensity and proliferative activity were negatively associated. In summary, oral administration of *B.S*-Dia to sows could lead to enhanced proliferation of T lymphocytes.

### Effects of *B.S*-dia on levels of cytokines and antiviral protein in sows’ colostrum

**Fig. 3:**
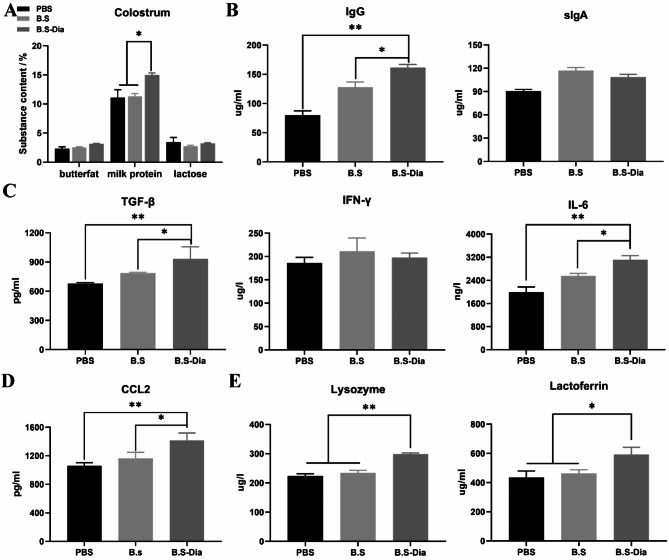
**(A)** Contents of milk fat, lactose, and milk protein in colostrum were analyzed using a milk composition analyzer; **(B-D)** IgA, IgG, cytokines, and CCL2 levels in colostrum were measured by ELISA; **(E)** Antiviral proteins in colostrum were evaluated by ELISA. Significant differences between different treatment groups were analyzed by one-way ANOVA, n = 5, *P < 0.05, **P < 0.01

The nutrients in the sows’ colostrum include mainly lactoprotein, milk fat, and lactose. Firstly, the proportions of these three components in the colostrum were measured. Compared with the *B.S* group (average = 11.3) and the PBS group (average = 11.0), the lactoprotein content in the colostrum from the sows of *B.S*-Dia group was significantly increased (*P* < 0.05), with an average increase of 15% (Fig. 3A). In addition to provide nutrition from casein, the lactoprotein in the colostrum also includes antibodies, cytokines, and antiviral substances. It was found that administration of *B.S*-Dia resulted in elevated levels of IgG in the colostrum (average = 161.1), significantly differing from the *B.S* and the PBS groups (*P* < 0.05, *P* < 0.01, respectively) (Fig. 3B). The concentrations of IFN-γ, TGF-β, and IL-6 in colostrum were then evaluated as these cytokines have antiviral functions. It was found that the administration of *B.S*-Dia resulted in elevated concentrations of TGF-β (average = 933.1) and IL-6 (average = 3112.2) in the colostrum, compared with the other groups (*P* < 0.05 and *P* < 0.01, respectively) while there were no significant differences in the IFN-γ concentrations between the different groups (Fig. 3C). The CCL2 contents increased significantly in the *B.S*-Dia group compared with other groups (*P* < 0.05, *P* < 0.01) (Fig. 3D). Additionally, the concentrations of the two important antiviral proteins, lysozyme and lactoferrin, were observed to be 298.9 µg/L and 592.0 µg/mL, respectively, in the colostrum (*B.S*-Dia group), with the levels of both proteins significantly higher than those in the *B.S* group (lysozyme, average = 243.3, lactoferrin, average = 63.1) and PBS group (lysozyme, average = 233.7, lactoferrin, average = 435.9) (Fig. 3E).

**Fig. 4:**
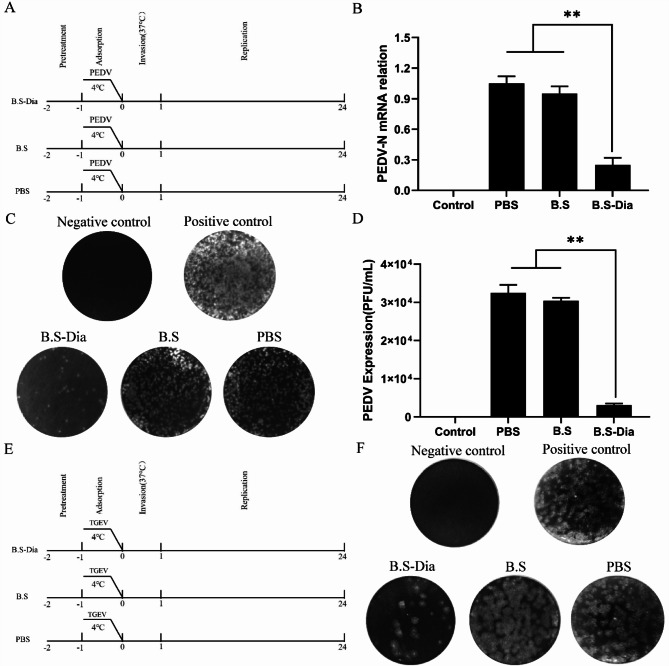
**(A)** Anti-PEDV properties of whey after oral administration of *B.S-*Dia, *B.S*, and PBS. PBS, whey from sow colustrum in the PBS group was used to treat cells; *B.S*, cells pretreated with colostral whey in the *B. subtilis* group; *B.S*-Dia, the colostral whey from sows in *B.S*-Dia group was used to treat cells. RT-qPCR **(B)** and plaque tests (**C** and **D**) were used to assess antiviral effects. Differences between different treatment groups were assessed by one-way ANOVA, **P < 0.01. (E) Anti-TGEV effects of whey in the *B.S-*Dia, *B.S.*, and PBS groups. **(F)** Plaque tests were used to assess the antiviral effects

As shown in Supplementary Fig. 2A, the whey collected from sows in the *B.S*-Dia, *B.S*, and PBS groups was preincubated with virus prior to PEDV infection and the mixture was subsequently inoculated into Vero E6 cells. The results of the plaque assay (Supplementary Fig. 2B, C) showed that the whey in the *B.S*-Dia, *B.S*, and PBS groups pretreated with PEDV failed to prevent PEDV infection. On the contrary, the whey that had been preincubated with Vero E6 cells prior to viral infection, then infected with PEDV (Fig. 4A). The RT-qPCR (Fig. 4B) and plaque assay (Fig. 4C, D) results suggested that whey from sows fed with *B.S*-Dia-pretreated cells significantly inhibited intracellular both viral expression and the release of infectious particles. Next, to investigate the effect of antiviral substances in the colostrum on other enteroviruses, a similar experiment was performed (Fig. 4E), and the results showed that whey from sows fed with *B.S*-Dia-pretreated cells also significantly inhibited transmissible gastroenteritis virus (TGEV) infection (Fig. 4F).

### Effect of congenital lactogenic immunity on PEDV infection in newborn piglets

**Fig. 5:**
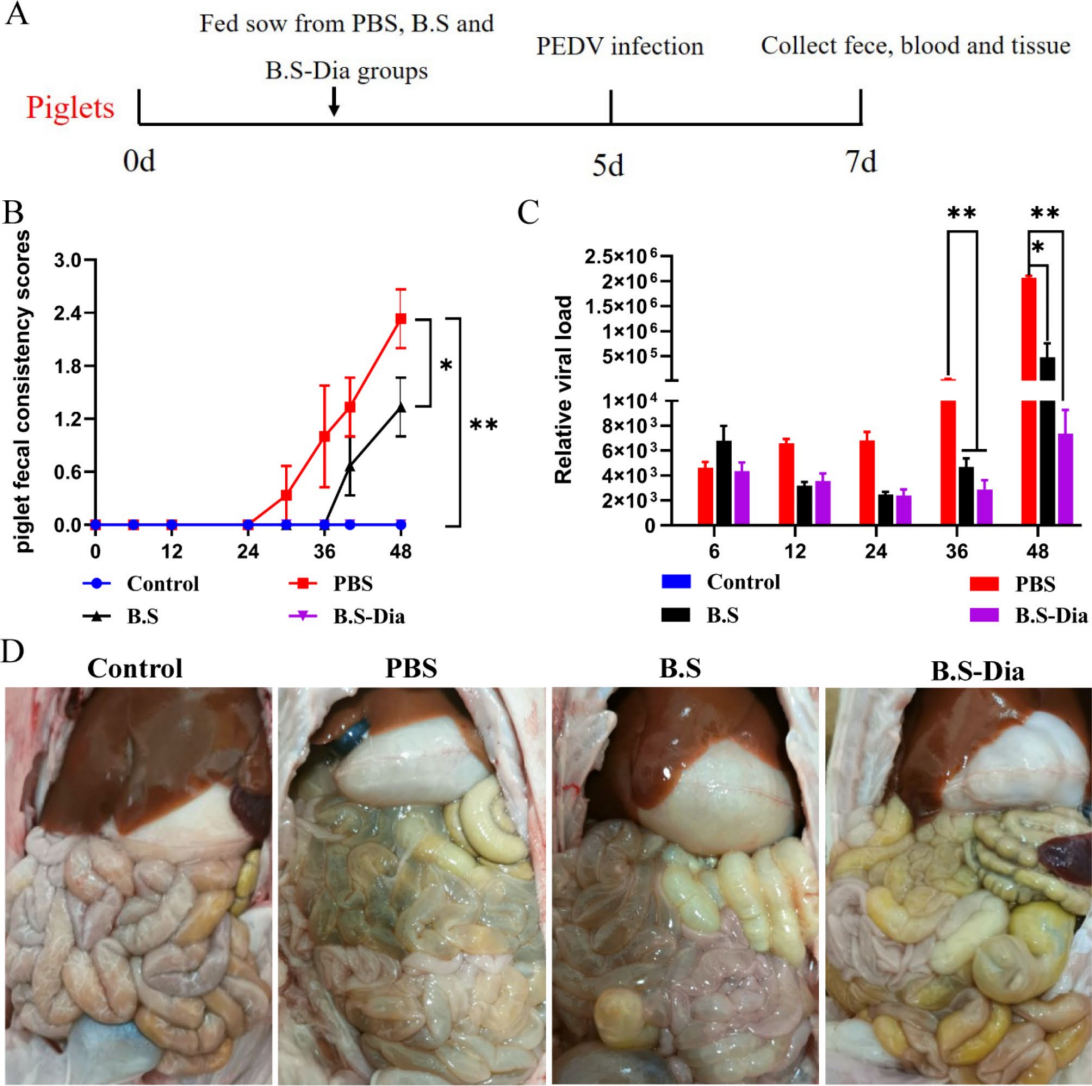
**(A)** Schematic diagram showing oral infection of piglets with PEDV; **(B)** Piglet feces were scored by consistency, n = 5; **(C)** Viral loads in feces were measured by RT-qPCR, n = 5; **(D)** Clinical symotoms in piglets. Statistical analysis was performed using the two-way ANOVA with repeated measures and Bonferroni’s correction for multiple comparisons, n = 5, *P < 0.05, **P < 0.01

To evaluate the effects of congenital lactogenic immunity (Fig. 5A), the clinical symptoms of piglets infected with PEDV were monitored. The results revealed that piglets in the PBS and *B.S* groups displayed typical PEDV symptoms at 48 h, including acute watery diarrhea, depression, and decreased appetite, but these symptoms were absent in piglets from the *B.S*-Dia group. To evaluate the piglets’ diarrhea, the stool consistency was scored, with higher scores indicating more severe diarrhea. The piglets in the PBS group had a diarrhea score close to 2.4 at 48 h after infection with PEDV, which was significantly higher than those in the *B.S* (1.2) and *B.S*-Dia (0) groups (Fig. 5B). Moreover, the detection of the viral load in feces showed that piglets of the *B.S*-Dia group had lower viral loads at 36 and 48 h after infection compared with the other groups (Fig. 5C). Additionally, the piglets in the *B.S*-Dia group exhibited no obvious pathological symptoms upon dissection and the piglets had abundant intestinal contents with no changes in the intestinal wall (Fig. 5D). However, piglets from the *B.S* and PBS groups had no intestinal contents, and the intestinal wall was relatively thin and transparent (Fig. 5D).

**Fig. 6:**
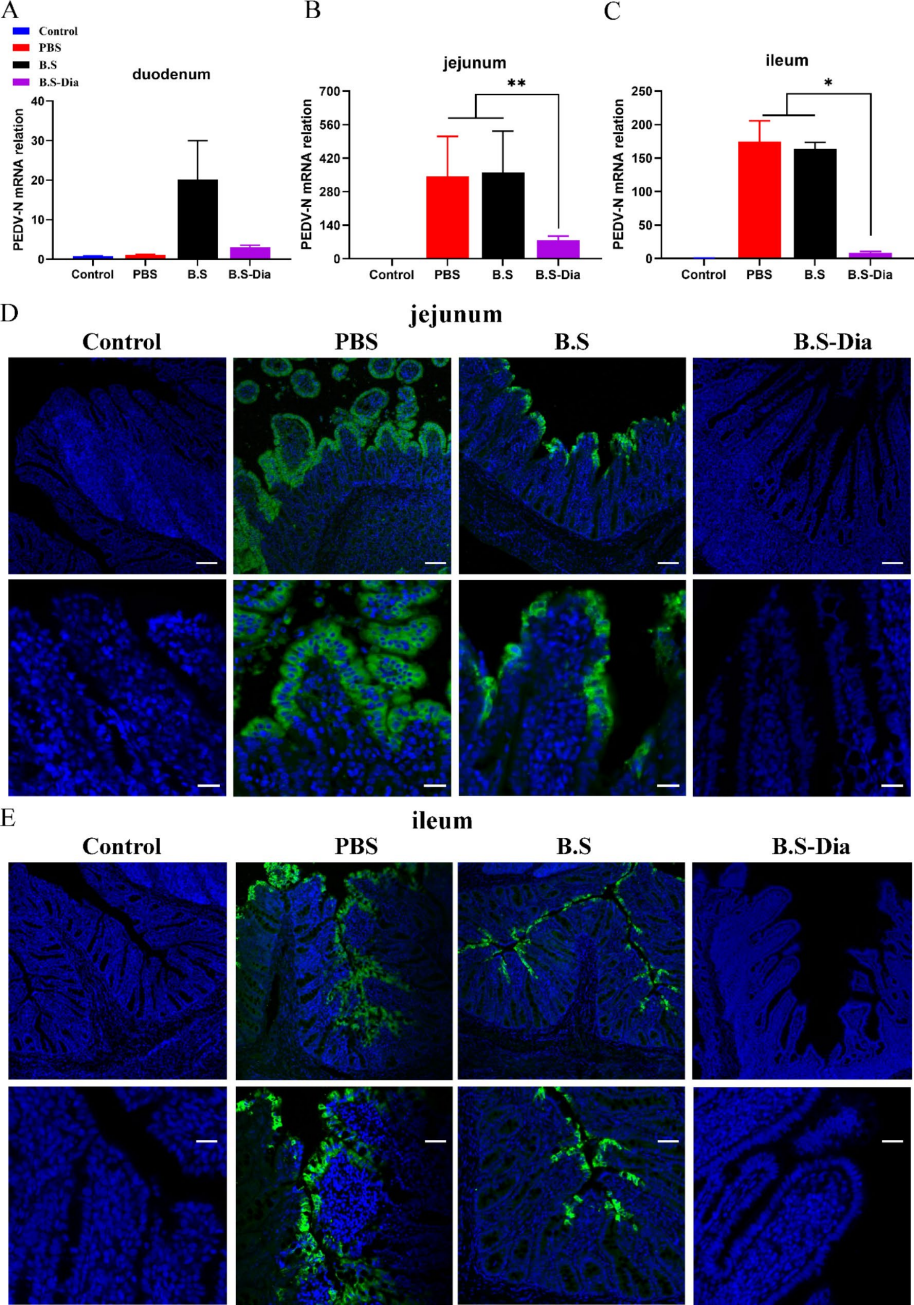
**(A-C)** Viral loads in the duodenum, jejunum, and ileum of piglets were measured by RT-qPCR, and differences between the different treatment groups were analyzed by one-way ANOVA, n = 5; **(D-E)** Viral loads in the jejunum and ileum of piglets were verified by immunofluorescence; Original magnification×100, ×400. scale bars = 50, 20 μm, *P < 0.05, **P < 0.01

The viral distribution in the intestinal tissue of the piglets was measured by RT-qPCR. It was found that PEDV was present primarily in the jejunum and ileum of the piglets, with significantly lower viral contents in the *B.S*-Dia group compared with the *B.S* and PBS groups (Fig. 6A-C). Moreover, immunofluorescence analysis showed that that there was no PEDV antigen in the jejunum and ileum villous epithelial cells of piglets in the *B.S*-Dia group, whereas a large number of PEDV-positive cells were found in the jejunum and ileum of piglets in the *B.S* and PBS groups (Fig. 6D-E). The above results indicated that congenital lactogenic immunity induced in sows by administration of *B.S*-Dia effectively reduced the PEDV viral loads in the piglets’ intestines.

### Effects of *B.S*-dia on piglet immunity

**Fig. 7:**
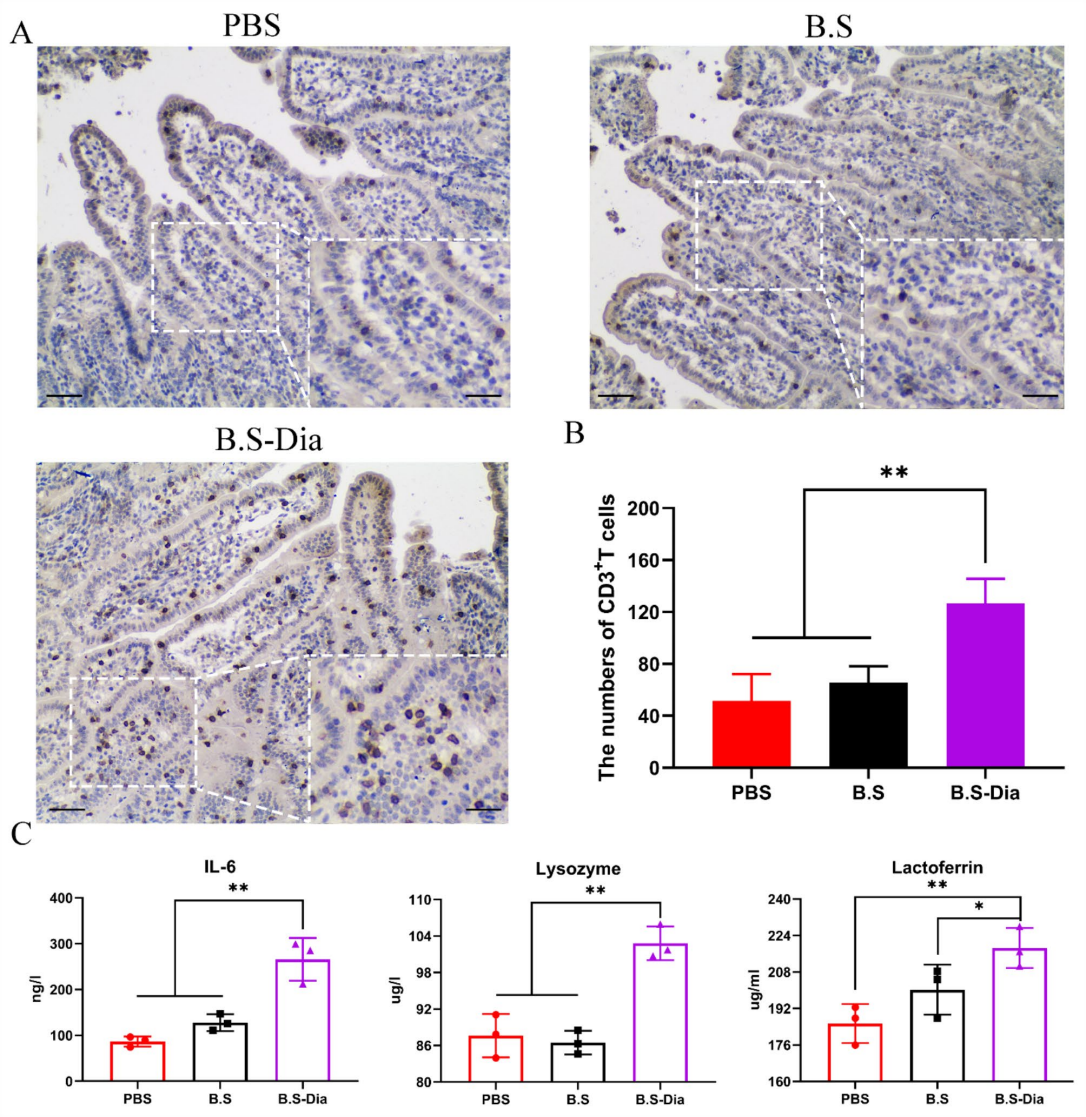
**(A)** Immunohistochemical assessment of lymphocyte (CD3) numbers in the intestinal tract of piglets, magnification×100, ×400, scale bar = 50 μm; **(B)** The numbers of CD3^+^T cells were counted in random fields (×400) and differences were analyzed by one-way ANOVA, **P < 0.01. **(C)** The detection of cytokines and antiviral proteins in the blood of piglets, and differences between the different treatment groups were determined by one-way ANOVA, *P < 0.05, **P < 0.01

Newborn piglets acquire immunity primarily through the colostrum, allowing them to resist infection by pathogenic microorganisms. In order to verify the effects of sow subject to oral *B.S*-Dia administration on piglets’ immunity, the number of lymphocyte (CD3) and the concentrations of IL-6, lysozyme, and lactoferrin in the piglets were investigated. The IHC results showed that the number of CD3 + T lymphocytes in the jejunums of piglets from the B.S-Dia group was significantly increased (average = 127), while the number of CD3^+^T lymphocytes in the other groups was lower (Fig. 7A, B). Additionally, compared with the PBS and *B.S* groups, the levels of IL-6, lysozyme, and lactoferrin in the serum of piglets from the *B.S*-Dia group were significantly increased (Fig. 7C). In summary, oral administration of *B.S*-Dia to sows significantly enhanced the immunity of the newborn piglets.

## Discussion and conclusions

PED is a major cause of diarrhea in newborn piglets and has high levels of morbidity and mortality [21]. Although specific vaccines given to the sows to provide lactogenic protection for the newborn piglets, the mutation rate of PEDV lags behind the development of new vaccines, reducing the effectiveness of PED prevention and control. A previous study found that high levels of specific antibodies could be produced in sows’ milk after oral administration of a PEDV vaccine, although the piglets still developed diarrhea symptoms after challenge, possibly resulting from infection with different subtypes of PEDV (different from the vaccine strains) [18]. These results indicate that the cross-protective effects of different vaccine strains are not ideal. Surprisingly, our results revealed that oral administration of *B.S*-Dia could not only increase the number of immunocytes in the colostrum, but also modulated the levels of cytokines levels and antiviral proteins in the colostrum. Importantly, congenital lactogenic immunity effectively prevented PEDV infection in the piglets. Additionally, the innate immunity was not limited to viral antigens and might thus protect the piglets from infection with other enteroviruses, such as TGEV. However, the cytokines and antiviral proteins associated with congenital immunity are only present in the piglets for a short time and are not able to provide long-term protection against infection.

Dendritic cells (DCs) are widely distributed in the intestinal mucosa of pigs and are able to extend dendrites across the epithelium into the intestinal lumen to capture *Bacillus subtilis*. The DCs then migrate to mesenteric lymph nodes, thereby activating naive T lymphocytes and promoting cytokine secretion. A previous study demonstrated that oral administration of *B.S*-Dia could induce the maturation of mouse bone-derived DCs and enhance proliferation of T lymphocytes [10]. Finally, T lymphocytes, cytokines, and antiviral protein migrate to the mammary gland through the gut-mammary gland-SIgA axis and enter the colostrum [22, 23]. Therefore, feeding with *B.S*-Dia effectively stimulated the production of congenital lactogenic immunity.

On day 40 of pregnancy in pigs, the numbers of T lymphocytes in the mammary gland began to increase gradually and reach a maximum on the 80th day of pregnancy [2]. Hence, *B.S*-Dia was administered on the 80th day of gestation, and was repeated several times until parturition. The results showed that the numbers of T lymphocytes in the colostrum increased, together with significant increases in their proliferation. Moreover, the chemokine CCL2 has been shown to promote the migration of T lymphocytes across mammary gland epithelial cells [14]. It was also observed that oral administration of *B.S*-Dia resulted in significantly increased levels of CCL2 in the colostrum, which could be the main reason for the increase in T lymphocyte numbers in the colostrum. T lymphocytes in the colostrum are able to pass through the gut of newborn piglets and enter the circulatory system, thus contributing to the development of the immune system [24]. In addition to T lymphocytes, colostrum also contains a large number of monocytes, which differentiate into the macrophages after entering the piglet intestine [25]. Viral infection can lead to the formation of phagolysosomes in macrophages [26] where hydrolases degrade the virus and recycle the components through exocytosis. It is beneficial for piglets to resist viral infection [26].

Cytokines are transferred to newborn piglets through the colostrum and play an important role in the regulation of immune and antiviral responses [27]. IL-6 can regulate the body’s immune response [28]. In addition, IL-6 also exhibits antiviral functions and has been shown to prevent the accumulation of hepatitis B virus in the cells, thereby effectively inhibiting viral replication [29]. IL-6 also suppresses viral invasion by downregulating the expression of HBV-specific receptors on the cell surface [30]. TGF-β affects cell proliferation and regulates intestinal inflammation, [31, 32]. Additionally, TGF‐β also exhibits diverse antiviral properties. In hepatocytes, TGF‐β was found to exert antiviral functions by blocking the invasion and replication of hepatitis C virus in a TGF‐β /SMAD signaling pathway-dependent manner [33]. Moreover, another study showed that TGF‐β could also promote IFN-β expression through modulation of the TGF‐β/SMAD signaling pathway to inhibit the replication of human syncytial virus in macrophages [34]. In this study, administration of *B.S*-Dia to sows was found to significantly enhance the antiviral capacity of piglets, which might be related to increased concentrations of cytokines.

Swine colostrum is relatively rich in lactoferrin, which is also able to inhibit viral replication [35, 36]. Lactoferrin blocks viral invasion essentially by binding to viral particles or adhesion molecules on the surfaces of target cells. It has been demonstrated that lactoferrin prevents entry of viruses by binding to rotavirus particles, thus preventing viral binding to specific receptors [37, 38]. Sano et al. found that lactoferrin could interact directly with the F1 subunit of the F protein from the respiratory complex virus, thereby reducing viral infection [39]. Lang et al. reported that lactoferrin blocked the interaction between the spike protein of SARS-CoV-2 and heparan sulfate proteoglycans on the surfaces of host cells [40]. In addition, colostrum also contains lysozyme which shows significant antiviral properties. Because lysozyme carries a strong positive charge, it can interact directly with negatively charged viral proteins to form viral DNA and RNA apo-groups double salt and inactivate the viruses [41]. In another study, Mann et al. demonstrated that the positive charges carried by lysozyme inhibited virus-induced cell fusion, thereby inhibiting the viral infection [42]. In our study, administration of *B.S*-Dia to sows elevated the concentrations of lysozyme and lactoferrin in the colostrum, which might benefit the newborn piglets against PEDV infection.

In general, pregnant sows fed with *B.S*-Dia were able to improve congenital lactogenic immunity and protect newborn piglets from PEDV infection, providing another pathway for the prevention of PED. However, there are limitations to this study. Firstly, it is not known which innate immune factors in the colostrum can inhibit viral replication remains unknown,and future studies are warranted to better understand the mechanisms involved. Furthermore, *B.s*-Dia needs to be tested in larger samples in different pig farms to further verify the effect of congenital lactogenic immunity.

### Electronic supplementary material

Below is the link to the electronic supplementary material.

Supplementary Material 1

Supplementary Material 2

Supplementary Material 3

## Data Availability

The data that support the findings of this study are available from the corresponding author upon reasonable request.
