# Supplementary figures and images for: Feeding with 4,4′-diaponeurosporene-producing Bacillus subtilis enhances the lactogenic immunity of sow
Source: BMC Vet Res. 2023 Dec 19;19:280. doi: 10.1186/s12917-023-03846-3 (PMC10729370; doi:10.1186/s12917-023-03846-3)

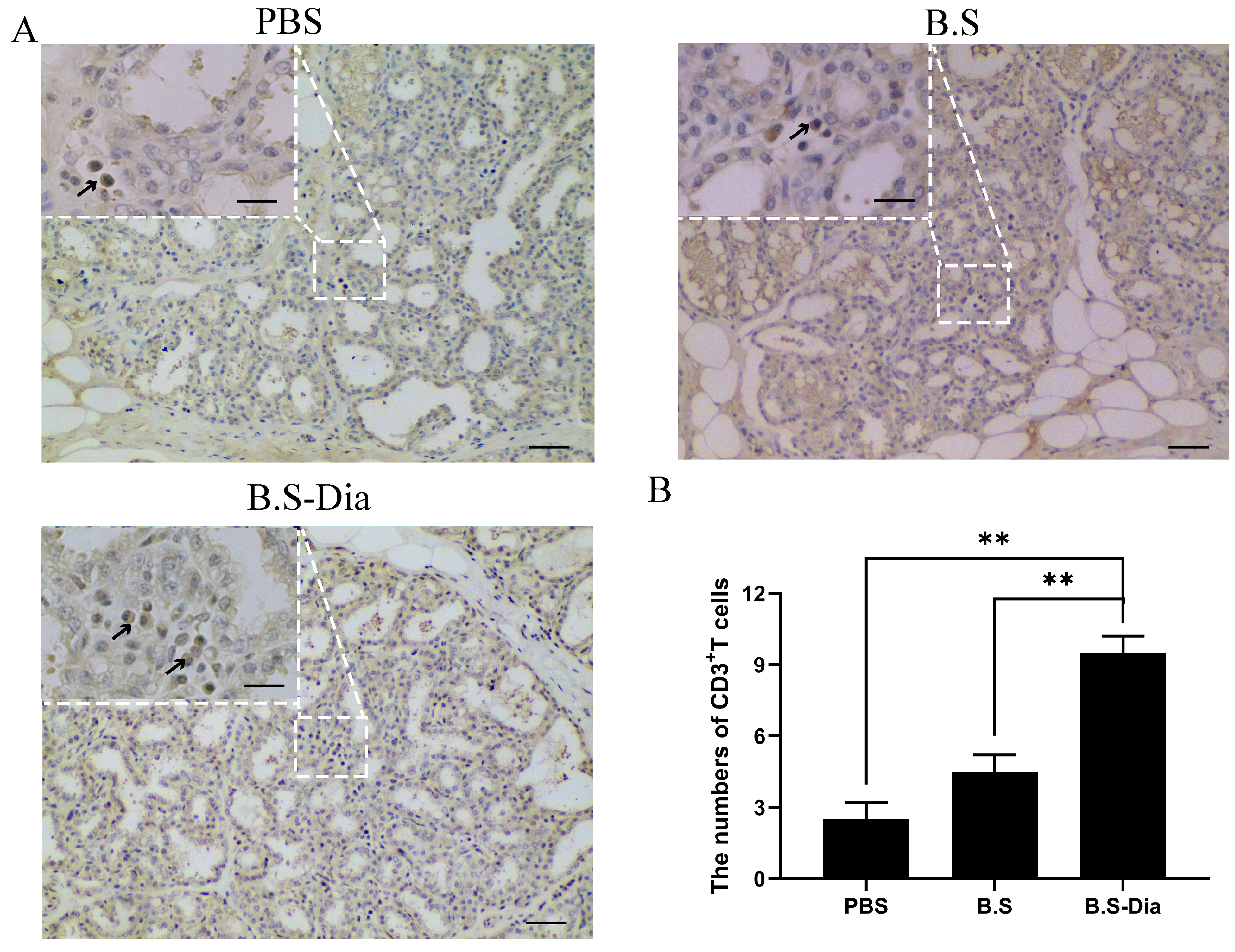

Supplement: Supplementary file 1 — Supplementary Material 1 [file 12917_2023_3846_MOESM1_ESM.tif]

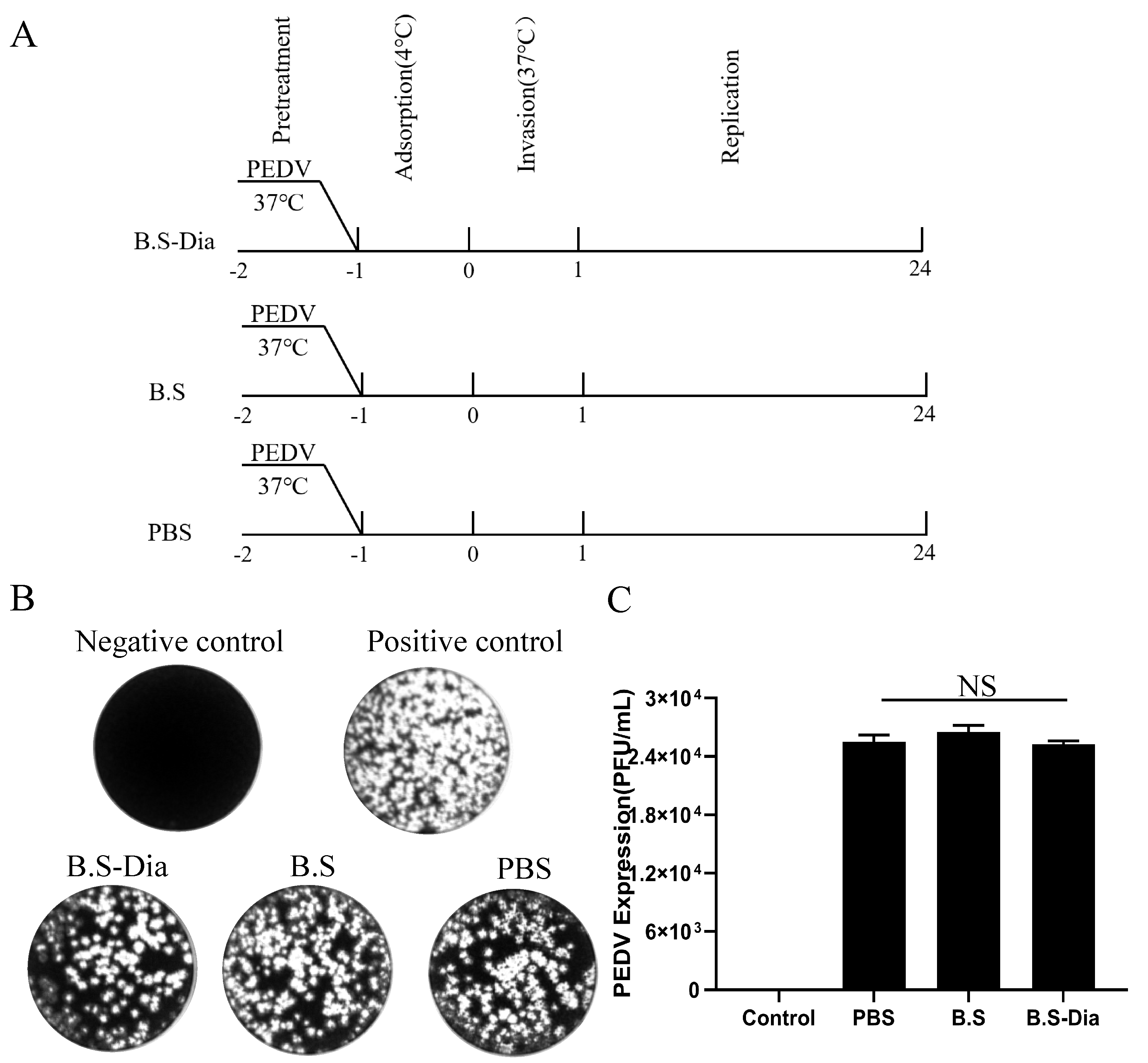

Supplement: Supplementary file 2 — Supplementary Material 2 [file 12917_2023_3846_MOESM2_ESM.tif]
