# Supplementary material for: Feeding with 4,4′-diaponeurosporene-producing Bacillus subtilis enhances the lactogenic immunity of sow
Source: BMC Vet Res. 2023 Dec 19;19:280. doi: 10.1186/s12917-023-03846-3 (PMC10729370; doi:10.1186/s12917-023-03846-3)
Supplement: Supplementary file 3 — Supplementary Material 3 [file 12917_2023_3846_MOESM3_ESM.docx]

**Supplementary Fig1** (A) Immunohistochemical detection on the number of lymphocytes (CD3) in the mammary gland, n=3; Magnification×100, ×400, scale bar=50, 20μm. (B) The numbers of CD3^+^T cells were counted in random fields (×400), and the significant difference between different treatment groups was determined by one-way ANOVA, ***P*<0.01.

**Supplementary Fig2** (A) Anti-PEDV effects analysis of sow whey after oral administration of *B.S-*Dia, *B.S* and PBS. PBS, the colostral whey from sows in the PBS group was pretreated with PEDV; *B. S*, PEDV pretreated with colostral whey of sows in oral administration of *B. subtilis* group; *B.S*-Dia, the colostral whey from sows in *B.S*-Dia group was pretreated with PEDV. (B) Plaque tests were used to detect the antiviral effect. (C) The significant difference between different treatment groups was determined by one-way ANOVA, NS, with no significant difference.
